# Understanding Antecedents and Attributes in the Context of Upward Referral for Obstetric Emergencies: A Grounded Theory Study

**DOI:** 10.12688/f1000research.174876.3

**Published:** 2026-03-02

**Authors:** Final Zimkhitha Juqu, Dr Esther Lydie Mbobnda Kapcheb, Dr Zamadonda Nokuthula Xulu-Kasaba, Prof. Olivia Baorapetse Baloyi

**Affiliations:** 1 School of Medicine, College of Health Sciences, University of KwaZulu-Natal, Durban, KwaZulu-Natal, 4041, South Africa; 2 Department of Basic Medical Services, Durban University of Technology, Durban, KwaZulu-Natal, 4041, South Africa; 3 Department of Optometry, College of Health Sciences, University of KwaZulu-Natal, Durban, KwaZulu-Natal, 4041, South Africa; 4 Department of Nursing, Walter Sisulu University Faculty of Health Sciences, Mthatha, Eastern Cape, South Africa

**Keywords:** Community Health Centers, Healthcare Facilities, Maternal Mortality, Obstetric Emergencies, Upward Referral

## Abstract

**Background:**

Reducing maternal and neonatal deaths in low- and middle-income countries requires a comprehensive understanding of the challenges within the healthcare system. Effective upward referral systems play a crucial role in ensuring timely emergency care for obstetric complications in the healthcare system. The aim of this study is to understand the factors (antecedents) and defining characteristics (attributes) that influence upward referrals of obstetric emergencies. from Community Health Centres to higher-level hospitals in the OR Tambo district, Eastern Cape, South Africa.

**Methods:**

Data were collected through document analysis, observations, focus group discussions and in-depth interviews, consisting of 59 participants, using a qualitative grounded theory approach.

**Results:**

The study reveals a complex web of interrelated antecedents and attributes that influence the effectiveness of upward referrals in obstetric emergencies. While formal protocols exist, referral decisions are often undermined by systemic barriers. The findings suggest that the lack of coordination between referring and receiving facilities, coupled with fragmented feedback systems, hampers continuity of care. Despite these challenges, instances of effective referral were observed in settings where interprofessional collaboration, timely information sharing, and adequate preparation of patients were prioritised.

**Conclusion:**

Effective upward referral of obstetric emergencies depends on timely decision-making, adequate patient preparation, clear communication, and inter-facility collaboration. This highlights the need for an integrated approach that strengthens health system functions, which are essential to reducing preventable maternal and neonatal morbidity and mortality in resource-constrained settings like South Africa.

**Glossary**
CHC: Community Health Centres
ECP: Emergency Care Practitioner
EMS: Emergency Medical Services
FGD: Focus group discussions
GT: Grounded Theory
HIC: High-income countries
IDI: Individual in-depth interviews
LMIC: Low- and middle-income countries
MMR: Maternal Mortality Rate
MO: Medical Officer
MOs: Medical officers
MW: Midwife
NCCEMD: National Committee for Confidential Enquiry into Maternal Deaths
NdoH: National Department of Health
OR: Oliver Reginald
SADAG: South African Depression and Anxiety Group
SSA: Sub-Saharan Africa
WHO: World Health Organization

## 1. Introduction and background

Maternal and neonatal morbidity and mortality remain a significant public health issue in the world. ^1^ Annually, approximately 287 000 women and 2.3 million newborns die due to preventable obstetric emergencies. ^1^ A significant portion of nearly two-thirds of these global maternal deaths occur in Sub-Saharan Africa (SSA), many of which could be prevented with timely emergency care. This statistic draws attention to persistent healthcare inequities that restrict access to quality maternal health services. ^1^ To address this issue, the WHO advocates for effective referral systems to reduce maternal and neonatal morbidity and mortality. Kanyesigye et al. ^2^ echo this sentiment, emphasising that timely access to specialised care through upward referral is vital in obstetric emergencies.

High-income countries (HICs), such as the United Kingdom ^3^ and Scandinavian nations ^4^ are some of the countries that benefit from effective, well-established upward referral systems. Their systems are supported by clear guidelines, efficient emergency transport systems, network connectivity, and advanced medical technology. ^5^ In contrary, many low- and middle-income countries (LMICs), including South Africa, face substantial challenges in developing and maintaining effective upward referral systems. ^6^ These challenges often involve healthcare system limitations ^7^ that contribute to high maternal and neonatal morbidity and mortality rates resulting from obstetric emergencies. ^8^ This is despite substantial evidence that managing obstetric emergencies within an effective upward referral system is paramount in maternal healthcare. ^9^ Common obstetric emergencies such as postpartum haemorrhage, pre-eclampsia, cord prolapse, and obstructed labour require instant recognition, action and referral to ensure that favourable maternal and neonatal health outcomes are achieved. ^10^

In South Africa, Community Health Centres (CHCs) serve as the first point of contact for pregnant women. CHCs are led by midwives, who are the primary caregivers responsible for identifying and managing obstetric emergencies ^11^ and play a key role in initiating upward referrals. ^6^ However, in provinces such as the Eastern Cape, healthcare infrastructure is often inadequate, with CHCs lacking essential resources, equipment and trained staff necessary to manage complex obstetric emergencies. Additionally, the geographical isolation of many communities in this province further complicates timely referrals, as factors such as long distances and poor road conditions delay the transfer of patients to higher-level healthcare facilities. ^12^ These structural barriers systematically hinder the midwives’ crucial role in managing obstetric emergencies and initiating upward referrals. ^11^

The challenges faced by South African midwives working in CHCs mirror those observed in other LMICs. Countries such as Ethiopia, Uganda and Nigeria face comparable issues, including limited access to emergency transport, poor communication with referral hospitals and bureaucratic delays, all of which adversely affect maternal health outcomes. ^9^ Yet, midwives’ ability to assess the severity of obstetric complications and initiate timely referrals is critical to reducing maternal and neonatal mortality. ^13^ However, systemic barriers continue to hinder the effective implementation of these practices.

This manuscript explores the antecedents and attributes of “effective upward referral” for women with obstetric emergencies from CHCs to higher-level care in the OR Tambo district. It examines healthcare practitioners’ perceptions, the causal conditions or antecedents (including contextual factors) and the essential attributes influencing effectiveness. While the broader study uses a Grounded Theory design, this paper focuses solely on antecedents and attributes, without presenting the full emergent model. By addressing these factors, the study contributes to improving maternal and neonatal health, aligning with Sustainable Development Goal 3 (Good Health and Well-being), particularly targets 3.1 (specifically the targets of reducing maternal mortality), 3.2 (ending preventable newborn deaths) and 3.8 (and achieving universal access to quality healthcare services). ^14^

## 2. Methodology

### 2.1 Design, approach and paradigm

Underpinned by a social constructivist paradigm, ^15^ this study employed a qualitative approach to explore how midwives, paramedics and medical officers (MOs) experience the phenomenon of upward referral of obstetric emergencies in their natural work settings. While the broader study followed a Grounded Theory design, ^16^ its use here is limited to its analytic strengths, guiding the systematic identification of antecedents and attributes without presenting the full emergent theory.

### 2.2 Setting and context

Despite a decrease in maternal deaths in healthcare facilities in the Eastern Cape, from 138 per 100 000 live births in 2017 to 128.9 per 100 000 in 2022, ^17^ districts with referral hospitals continue to experience challenges. Among these districts, OR Tambo is the most challenged district with the highest Maternal Mortality Rate (MMR), largely attributed to the presence of a highly specialised central hospital, within the district. ^6^

Within this district, a major sub-district which includes five Community Health Centres (CHCs), carries a disproportionately high MMR of 378.5 deaths per 100 000 live births. ^18^ Referral pathways in this area are often strained due to wide geographic coverage, limited transport infrastructure ^19^ and the urgent nature of obstetric emergencies. Accordingly, data collection for this study focuses on this high-burden sub-district to better understand and address the elevated maternal health challenges present.

### 2.3 Study participants

Purposive and theoretical sampling methods were employed to ensure that the selected participants could provide rich and meaningful data essential for understanding the phenomenon explored in this study. ^20^ Midwives, MOs and Emergency Medical Services (EMS) paramedics were purposively sampled for their key roles in managing and referring obstetric emergencies. In the sub-district that this study is focusing on, there are 128 midwives (CHCs and hospitals), 13 MOs and approximately 202 paramedics, with only 3 holding Emergency Care Technician or Emergency Care Practitioner (ECP) certifications. The theoretical sampling in this study did not rely on a predefined sample size; instead, it involved tracking the participants over time, guided by the theoretical concepts that emerged during data collection. To ensure the selected participants contributed relevant information, theoretical sampling was used to identify and address data gaps.

### 2.4 Data collection methods and process

Data were collected from August 2024 to May 2025 using focus group discussions (FGDs), individual in-depth interviews (IDIs), document analysis and observations. This multi-method approach strengthened methodological rigour and enabled systematic, constant comparative analysis to identify key antecedents and attributes of the phenomenon. ^20^ FGDs were conducted with midwives at 5 CHCs and hospitals. A total of thirteen FGDs were conducted comprising five at CHCs and eight at hospitals. Each FGD, facilitated in English and isiXhosa, lasted 35 minutes to an hour and involved three to five midwives, as recommended. ^21^ The FGDs were led by the primary researcher and co-facilitated by her supervisor. Additionally, the primary researcher conducted eight IDIs with MOs and three with paramedics, each lasting 35 to 45 minutes, conducted in English and isiXhosa. The 24 data collection sessions adhered to the suggested approach, ^22^ which emphasise that saturation should prioritise conceptual completeness and relational depth across categories, rather than rigid sample sizes. Data collection continued until theoretical saturation was achieved, indicated by no emergence of new categories or properties and confirmed relationships among existing concepts, as guided by the iterative principles of theoretical sampling. ^22, 23^ The FGDs and IDIs were audio recorded in distraction-free settings, at times convenient to participants. To further enrich the data, a triangulation approach was used, which included non-participant observations and document analysis, guided by an observation and a document analysis guide. These methods provided valuable insights into the upward referral process for obstetric emergencies, revealing both commonalities and differences in the perspectives of midwives and MOs.

### 2.5 Ethical considerations

Ethical principles were maintained throughout the study, beginning with obtaining ethical clearance from the University of Kwa-Zulu Natal (UKZN)’s Biomedical Research Ethics Committee BREC (reference number: BREC/00006633/2024), approved on the 22 ^nd^ of May 2024. Further approval was also secured from relevant authorities, including the Eastern Cape Department of Health, as well as key departmental heads, such as the District Manager and Sub-District Manager. Additionally, approval was obtained from the Chief Executive Officers and Operational Managers of the participating data collection sites. The researcher sought informed consent from participants, including permission for audio recording. Participants were reminded of their voluntary participation and right to withdraw without consequences. Strict confidentiality measures were implemented, ensuring all data remained anonymous and untraceable to individual participants.

Given the sensitive nature of obstetric emergencies, the researcher prioritised mitigating potential emotional or psychological distress, particularly when participants discussed adverse or life-threatening events, ^24^ such as experiences of loss or trauma in the referral process. The researcher proactively provided contact details for the South African Depression and Anxiety Group (SADAG) to all participants. No instances of distress requiring referral were reported. All data is managed with stringent confidentiality measures where it is stored on a password-protected computer, accessible only to the researcher and authorised supervisors. In compliance with data protection protocols, remaining electronic files will be permanently deleted five years after study completion.

### 2.6 Data analysis process

Data analysis followed a Straussian Grounded Theory approach ^20, 25^ using open, axial, and selective coding with constant comparative analysis. Interviews were transcribed verbatim by the researcher and research assistant and verified by the supervisor, co-supervisor, and co-authors to ensure accuracy. Analysis began with open coding, examining transcripts line by line to identify initial codes and patterns, with reflections captured through memoing. Axial coding explored relationships between categories, such as linking “clear communication” with “prompt decision-making,” while selective coding refined categories into a conceptual framework focused on antecedents and attributes, without presenting a full emergent theory. An independent coder used Atlas.ti to conduct inductive, line-by-line coding and organised the resulting codes deductively in alignment with the study’s objectives. Coding decisions were compared with those of the primary researcher and any discrepancies were resolved through discussion with the supervisory team until consensus was reached. Rigour was ensured through reflexive thematic analysis by maintaining a transparent coding trail, grounding interpretations in the data, and applying credibility, dependability, and confirmability criteria. Throughout, constant comparative analysis allowed iterative comparison of codes across interviews, observations, and documents. The researcher applied theoretical sensitivity, approaching data without preconceived notions, consulting GT experts, and co-constructing meaning with participants, consistent with the social constructivist paradigm. This systematic process ensured that findings were firmly grounded in participants’ experiences.

### 2.7 Rigour

Trustworthiness was ensured following established criteria for credibility, dependability, confirmability and transferability ^26^ within the Straussian Grounded Theory framework. ^20^ Credibility was established through prolonged engagement with participants, iterative constant comparative analysis, and member checking, allowing emerging codes and categories to be validated and grounded in participants’ experiences. Dependability was supported by maintaining a transparent audit trail documenting all steps of data collection, coding, memoing, and category development, reflecting the systematic and iterative nature of GT. Confirmability was achieved through reflexive memoing, independent coding by the research team, consensus discussions on emerging categories, and investigator triangulation, ensuring that findings were derived from the data rather than researcher preconceptions. Transferability was addressed through rich contextual descriptions, detailed accounts of research objectives, methods, and the researcher’s role, enabling readers to understand how the identified antecedents and attributes may apply in similar settings. Multiple data sources and perspectives further strengthened triangulation, reinforcing the rigor and reflexivity central to GT methodology.

## 3. Findings

### 3.1 Profile of the participants

The study involved 59 participants each contributing valuable insights on aspects of the upward referral process for women with obstetric emergencies. The participants, consisted of midwives, MOs and paramedics. Participants ranged from different age groups with varying levels of professional experience, providing a diverse range of insights. All participant codes (e.g., FGD1_MW1) in Table 1 were assigned solely for organisational and analytic purposes and do not contain or derive from any personal identifiers. All data were de-identified in accordance with the Health Insurance Portability and Accountability Act (HIPAA) Safe Harbor method.

**Table 1.** Profile of the study participants, including professional role, gender and data collection method. Table 1 summarises the professional categories and roles of study participants involved in the upward referral of obstetric emergencies.

Group	Participant codes	Roles
CHC 1, FGD 1	FGD1_MW1 - FGD1_MW3	Female Midwives
CHC 2, FGD 2	FGD2_MW1 – FGD2_MW4	Female Midwives
CHC 3. FGD 3	FGD3_MW1 - FGD3_MW3	2 Male Midwives, 1 Female
CHC 4, FGD 4	FGD4_MW1- FGD4_MW4	Female Midwives
CHC 5, FGD 5	FGD5_MW1 - FGD5_MW3	Female Midwives
Hospital 1, FGD 6	FGD6_MW1 – FGD6_MW5	Female Midwives
Hospital 2, FGD 7	FGD7_MW1 - FGD7_MW3	Female Midwives
Hospital 2, FGD 8	FGD8_MW1 - FGD8_MW4	1 Male Midwife, 3 females
Hospital 3, FGD 9	FGD9_MW1 – FGD9_MW3	1 Male Midwife, 2 Females
Hospital 3, FGD 10	FGD10_MW1 – FGD10_MW4	Female Midwives
Hospital 1, FGD 11	FGD11_MW1 – FGD11_MW4	2 Male Midwives, 2 Females
Hospital 2, FGD 12	FGD12_MW1 – FGD12_MW4	Female Midwives
Hospital 3, FGD 13	FGD13_MW1 – FGD13_MW4	3 Female Midwives, 1 Male
Medical Officers	IDI_MO1 – IDI_MO8	5 Male, 3 Females
EMS Paramedics	IDI_PARAM1 - IDI_PARAM3	2 Female, 1 Male

### 3.2 Antecedents for effective upward referral of obstetric emergencies

Antecedents are the conditions or factors that must exist before the upward referral process can occur effectively. ^27^ According to the healthcare practitioners, these elements set the stage for upward referral and are often system-level or preparatory in nature. Table 2 presents the key antecedents identified as critical for ensuring effective upward referral of obstetric emergencies from CHCs to higher-level healthcare facilities.

**Table 2.** Summary of the antecedents identified as critical for effective upward referral of obstetric emergencies from community health centres to higher-level healthcare facilities. The themes and sub-themes were derived from qualitative analysis of participant interviews and focus group discussions.

Theme	Sub-theme
Clear and Timely Communication	Communication of relevant information clearly and quickly
	Provision of accurate and concise patient details to the receiving facility
Adequate Preparation and Documentation	Thorough patient preparation
	Ready availability of patient documents
Resource and Human Resource Management	Efficient distribution of resources
	Sufficient number trained midwives available

*3.2.1 Clear and timely communication*

Data sources indicated that clear and timely communication is crucial for obstetric emergencies upward referral process to guarantee readiness of the receiving facility. Two primary categories were identified: (a) Communication of relevant information clearly and quickly and (b) Provision of accurate and concise patient details to the receiving facility.

*3.2.1.1 Communication of relevant information clearly and quickly*

Data sources mentioned that a seamless upward referral process of obstetrical emergencies is ensured by quick and clear communication, which prevents misunderstandings and unnecessary delays, as highlighted in the quotes below:

“ *We immediately make sure the receiving facility is aware of everything happening with the patient. We update them with accurate information to avoid delays and misunderstandings*” (FGD1_MW1, CHC 1).

“ *… I ensure that I communicate relevant and clear information, so the next person knows what to expect and avoid delaying the patient from receiving care*” (FGD3_MW3, CHC 3).

Although participants consistently emphasised the importance of clear, quick, and relevant communication, they also highlighted various barriers that hinder the timeliness of referrals. These include communication breakdowns, network issues, and unpaid telephone bills. These challenges represent contextual and structural constraints that disrupt the implementation of otherwise effective communication practices.

“ *There’s a breakdown in communication because the information from the paramedics differs from the other side which causes an issue when we receive patients”* (FGD9_MW3, Hospital 3).

“ *We sometimes have network issues. We cannot reach where we are referring to in time and it puts mother and baby at risk*” (FDG5_MW2, CHC 5).

*“Last year (2024), landlines were cut off because the department didn’t pay the bill. Imagine trying to coordinate an emergency referral when you can’t even call an ambulance”* (FGD13_MW3, Hospital 3).

*3.2.1.2 Providing accurate and concise patient details to the receiving facility*

The provision of accurate and concise patient details emerged as equally important in ensuring effective upward referral of obstetric emergencies. The participants alluded that the referring facility needs to provide the receiving facility with accurate and concise details of the patient. Participants expressed that timely communication facilitates adequate preparation at the receiving facility, helping to prevent delays in emergency care. The extracts support this view.

“ *As the referring facility, we make sure that the patient’s information is communicated correctly. We send vital details and any immediate needs, so they know why the patient was referred, which allows the hospital to prepare for their arrival*” (FGD4_MW1, CHC 4).

“ *Provision of details helps prepare properly and ensures that there is no delay in treatment once the patient arrives*” (FGD2_MW3, CHC 2).

*“It would be good for CHCs to provide us accurate information … this saves us time as the receiving hospital, by the time the patient arrives, we have prepared for them” (*IDI5_MO5) *.*

MOs and midwives at receiving facilities further stressed the importance of knowing how the patient was managed at the referring hospital before transfer which is crucial for ensuring continuity of care.

*“When the patient arrives, we need to know what’s been done so we can continue from there. It helps us take the right action straight away”* (FGD9_MW1, Hospital 3).

*“Sometimes documentation is inaccurate and incomplete which increases the complexity of managing emergency care” (*FGD13_MW1, Hospital 3).

*“I need to know if blood tests are completed, so I don’t waste time repeating. But sometimes, someone forgets to put a sticker on the blood, and then we don’t know which ones were done which is time consuming”* (IDI2_MO2).

However, the consistency and completeness of such details are often undermined by factors such as the absence of standardised communication protocols, inconsistent documentation systems and inadequate digital infrastructure, which compromise the reliability of information flow, even when staff are committed. This issue was confirmed by the primary researcher, who noted that patients are frequently referred with essential pre-referral information missing, incomplete, or inconsistently recorded. Table 3 below represents an extract from the document analysis illustrating this:

**Table 3.** Example of incomplete and inconsistently documented pre-referral information identified through document analysis of referral records. Extract from document analysis showing the frequency of incomplete and inconsistent pre-referral information across months. Patient identifiers are anonymised using initials; missing entries indicate that essential obstetric information was not recorded at the time of referral.

Month	January	February	February	February	February	March	March	March	April	May	June	July	August
Patient identifier:	Jan1	Feb1	Feb2	Feb3	Feb4	Mar1	Mar2	Mar3	Not recorded because there was no obstetric	May1	Not recorded because there was no obstetric	Not recorded because there was no obstetric	Aug1
Reason for referral, timing, urgency and the outcome of the referral	Reason- severe preeclampsia BP 188/110 Pulse- 111	Retained products of conception	Imminent eclampsia	Incomplete miscarriage, bleeding	Incomplete miscarriage	Severe preeclampsia	Eclampsia	Eclampsia		Severe preeclampsia			Per vaginal bleeding
Midwives’ notes	N/A	Gave her two units of syntocinon ringers lactate. Midwife flagged delay in ambulance arrival. The clinic didn’t have Cytotec as per notes	N/A	N/A	20 units of oxytocin in ringers’ lactate	Given magnesium sulphate	N/A	Given magnesium sulphate	Given magnesium sulphate	N/A	N/A	N/A	IV therapy with normal saline. No volume expanders in the clinic, inclusive of Cytotec. Patient continued to bleed amid the delays in ambulance arrival

*3.2.2 Adequate preparation and documentation*

Participants indicated that proper preparation and documentation ensures that the receiving facility has all the necessary information to provide the best possible care upon the patient’s arrival. Under this facet, two key domains were noted: a) Thorough initial patient preparation and b) Ready availability of patient documents.

*3.2.2.1 Thorough patient preparation*

Participants stated that thorough patient preparation involved ensuring that the patient receives initial treatment, such as putting up an intravenous (IV) line, ensuring that stat doses of emergency drugs are given, etc. The extracts below reflect participants’ views:

“ *Before we refer, we make sure the patient is stabilised as much as possible. If there’s bleeding, we put up an IV line and administer*” (FGD5_MW3, CHC 5).

“ *We make sure we give the patient immediate treatment required so that there’s no time wasted when the ambulance arrives*” (FGD3_MW1, CHC 3).

Participants at the receiving hospitals indicated that patients are sometimes not adequately prepared. Gaps in patient preparation and documentation are primarily rooted in structural challenges, particularly workforce constraints that limit the capacity of CHC midwives, who are often overburdened and required to juggle multiple roles. As a result, even when the intent to stabilise patients is present, preparation may fall short due to these broader structural barriers. The following extracts attest:

“ *Sometimes patients are not lined, have no catheter, yet they are referred as foetal comprised, so we need to start from scratch which takes extra time*” (FGD6_MW5, Hospital 1).

*“When you are the only one on duty, managing deliveries, postnatal care, and emergencies, it becomes overwhelming and hard to prepare patients thoroughly”* (FGD3_MW2, CHC 3).

*“CHCs don’t always have doctors on duty, so a nurse just writes a quick note, and we end up repeating tests and delaying treatment*” (FGD11_MW2, Hospital 1).

*3.2.2.2 Ready availability of patient documents*

Data sources emphasised the importance of including the patient’s medical history and current condition to ensure that critical information is available upon arrival, thereby preventing unnecessary delays. Their views are reflected below:

“ *Receiving patients with a full record, including their medical history and recent treatments, makes a huge difference. We’re able to start care immediately, which really helps in emergencies*” (IDI3_MO3).

“ *Clear and accurate documentation is key. If the referral includes everything we need, like medical history, lab results, and a clear reason for the transfer, it saves time”* (FGD12_MW2, Hospital 2).

The participants further noted that missing documentation and medical history lead to unnecessary delays and can worsen the patient’s condition in the healthcare facility, as reflected below:

“ *Sometimes when we receive patients, medical history is missing, which causes delays. Before referral, everything has to be recorded*” (FGD10_MW2, Hospital 3).

“ *… when we looked at her records, there was barely any monitoring or vital signs history, and no mention stabilisation. It seemed like they just sent her without trying to manage her condition, and we had to rush to stabilise her right away … which puts unnecessary strain on us and endangers the patient”* (FGD9_MW2, Hospital 3).

“ *It’s frustrating when the documents are not there. We have to waste time asking for information that should have been provided already. This delay is a matter of life and death in such emergencies*” (FGD10_MW1, Hospital 3).

*3.2.3 Resource and human resource management*

It emerged from the data that the seamless operation of an efficient upward referral process for women with obstetric emergencies and the provision of appropriate care is contingent upon the effective management of resources and human resources, within the context of the healthcare system’s infrastructure and capacity. Two areas emerged under this category: (a) Efficient distribution of resources and (b) Sufficient number of trained midwives available.

*3.2.3.1 Efficient distribution of resources*

Successful referrals depend on the effective use of human resources, transportation and medical equipment. Yet, systemic barriers, such as limited ambulance availability and inadequately equipped ambulances, under-resourced CHCs and unmet basic needs often disrupt the process. As explained below:

“ *An ambulance on standby is important for referrals, but we struggle with this because there aren’t enough ambulances*” (FGD1_MW2, CHC 1).

“ *For the past two years we have had 1 ambulance in the area whose sole priority was maternity and neonatal cases. However, if there is an MVA, all ambulances are sent*” (IDI10_PARAM1).

*“Basics like diapers or sanitary pads for women are unavailable. These are not luxury items; they’re essentials for postnatal care. But women are left bleeding on folded linen”* (FGD13_MW4, Hospital 3).

The primary researcher also observed that none of the CHCs had an ambulance on site, despite most being in remote locations. Additionally, participants emphasised the importance of proper equipment in ambulances to ensure that they are reliable for transfers:

“ *Transport that is well equipped is vital for the referral to be smooth*” (IDI8_MO8).

“ *The ambulances don’t have basics and that comprises the mother and baby’s safety*” (FGD7_MW3, Hospital 2).

Referral records from CHCs further illustrated the practical constraints facing the upward referral system. The table below highlights systemic inefficiencies, including inconsistent documentation, lack of functional communication tools (such as absence of work telephones) and delays in ambulance response times. Critically, many of these referrals involved obstetric emergencies such as severe preeclampsia, eclampsia, incomplete miscarriages and per vaginal bleeding, all of which require urgent and time-sensitive interventions. Table 4 below represents an extract from the document analysis illustrating this:

**Table 4.** Documented systemic and operational constraints affecting community health centre-to-hospital referral processes for obstetric emergencies. Extract from document analysis of CHC-to-hospital referrals, illustrating systemic constraints in the referral process, including incomplete documentation, missing patient information, and delayed communication. Patient identifiers are anonymised using initials; missing entries indicate absence of recorded obstetric information.

Month	January	February	February	February	February	March	March	March	April	May	June	July	August
**Patient identifier:**	Jan1	Feb1	Feb2	Feb3	Feb4	Mar1	Mar2	Mar3	Not recorded because there was no obstetric	May1	Not recorded because there was no obstetric	Not recorded because there was no obstetric	Aug1
**The reason for referral, timing, urgency and the outcome of the referral**	Reason- severe preeclampsia BP 188/110 Pulse- 111	Retained products of conception	Imminent eclampsia	Incomplete miscarriage, bleeding	Incomplete miscarriage	Severe preeclampsia	Eclampsia	Eclampsia		Severe preeclampsia			Per vaginal bleeding
**Mode of communication**	Personal cell phones (CHC does not have work telephone)												
**Mode of transport- (ALL AMBULANCE) Request time vs arrival time**	10:40 13:00	06:15 10:25	15:00 22:00	15:00 20:00	21:50 Ambulance didn’t arrive; they took their own transport	10:52 12:07	09:13 11:34	19:41 20:15	08:30 12:00				O8:54 12:40
**Place of referral**	CHC 3												
**Where was the case referred to?**	Hospital 2	Hospital 2	Hospital 2	Hospital 1	Hospital 2	Hospital 2	Hospital 2	Hospital 2	Hospital 2	Hospital 1	Hospital 2	Hospital 2	Hospital 2
**Was the referring facility the first contact?**	Yes	Yes	Yes	No	Yes	Yes	Yes	Yes	Yes	No	Yes	Yes	Yes
**The approximate distance from the referring facility**	CHC 3 to Hospital 2 = 33.1 kms (39 min) CHC 3 to **Hospital 1** = 56.2 kms (59 minutes)												
**Use the ambulance from the sending facility?**	No – sending facility has no ambulance												

Participants reported that ambulances were initially equipped with essential tools, but systemic operational failures, including poor maintenance, led to damage or disuse, as mentioned below: “ *Sometimes there are not even the necessary tools, such as advanced monitors and it jeopardises the patient condition.* *Even when we did have the big monitors, they end up broken”* (IDI9_PARAM1).

*“We’ve had ambulances break down mid-transfer. We don’t only need more vehicles, but properly maintained ones”* (FGD12_MW1, Hospital 2).

*3.2.3.2 Sufficient number of trained midwives available*

In addition to resource allocation, having an adequate number of trained midwives is essential for effectively managing referrals of obstetric emergencies. Inadequate staffing and trained midwives may cause delays, which jeopardise the mother’s safety and makes the referral process harder. Participants highlighted the importance of adequate staffing:

“ *Managing emergency situations effectively and staying within our scope of practice requires an appropriate number of advanced midwives. When we’re short-staffed, there are delays and it’s harder to manage safe referrals*” (FGD8_MW1, Hospital 2).

“ *You focus on multiple cases alone because we are few and that is wrong when giving care*” (FGD6_MW3, Hospital 11).

The ability to deliver prompt and efficient care during referrals is directly correlated with staffing levels, as these statements demonstrate.

“ *For the referral of women in complication to be better, changes have to be made. We need more advanced midwives*” (FGD8_MW2, Hospital 2).

“ *It starts with improving primary health care. If facilities had more advanced midwives, at CHCs, it would make a big difference*” (FGD11_MW4, Hospital 1).

*“We need more hands-on deck, especially when we’re handling several emergency cases at once”* (FGD13_MW3, Hospital 3).

The primary researcher observed that staffing challenges in some facilities required a single midwife to cover multiple departments simultaneously, including the maternity ward, antenatal care, postnatal care, and family planning. As a result, midwives had to divide their attention and resources, shifting focus from managing emergencies to performing routine checks and providing counselling. This common operational reality highlights how institutional limitations, rather than deficits in clinical knowledge, constrain the midwife’s ability to ensure timely referral and safe patient preparation.

### 3.3 Attributes of effective upward referral of obstetric emergencies

Attributes, as defined ^27^ are the essential characteristics that constitute a concept. In the context of this study, attributes refer to the defining features of upward referral in obstetric emergencies. Table 5 below provides a summary as conceptualised from the perspectives of the data sources, illustrating how the phenomenon of ‘upward referral of obstetric emergencies’ was understood in this study.

**Table 5.** Summary of the defining attributes of effective upward referral of obstetric emergencies as conceptualised from participants’ perspectives. The themes and sub-themes were derived from qualitative analysis of participant interviews and focus group discussions.

Theme	Sub-themes
Timely and Effective Decision-Making	Timely and decisive action
Effective Collaboration and Teamwork	Trust in Teamwork
	Collaboration between Referral and Receiving Hospital Teams
Effective Referral Outcomes	Safe and stable transfer of the patient
	Timely arrival at the referral hospital
Post Referral Feedback and Continuous Improvement	Post Referral Feedback
	Review of referral cases for continuous improvement

*3.3.1 Timely and effective decision-making*

Effective prompt and timely decision-making in the process of upward referral of obstetric emergencies emerged as the cornerstone characteristic of ensuring safe and efficient transfers of patients needing advanced care. This attribute directly impacts the success of the referral process by guaranteeing that patients receive appropriate care without preventable delays.

*3.3.1.1 Timely and decisive action*

Timely and decisive action emerged as key for effective upward referral in obstetric emergencies. According to the data, when transferring patients to a higher level of care, prompt and efficient decision-making is crucial to ensuring they receive appropriate care as quickly as possible, as indicated below:

“ *In emergencies, every minute counts. When the patient’s life is at risk, the call for transfer should be made right away*” (FGD7_MW2, Hospital 2).

*“When we see danger signs, we refer immediately. But sometimes the ambulance takes hours”* (FGD6_MW1, Hospital 1).

While participants emphasised the importance of timely action, delays in ambulance response and other systemic obstacles frequently disrupted this ideal. They described having to make instant decisions about how to manage the patient’s condition while rapidly assessing risks. Field observations likewise revealed how healthcare workers assessed patients under pressure and initiated referrals with urgency. This is illustrated in the participants examples below:

*“When the mother presents with severe complications like a prolapsed cord, or pre-eclampsia, that we can’t manage on our own, we don’t have the luxury of time. We have to make a decision immediately and initiate an urgent referral*” (FGD2_MW1, CHC 2).

*“With fewer experienced doctors, decisions are either delayed or mishandled”* (IDI6_MO6).

*3.3.2 Effective collaboration and teamwork*

Effective collaboration and teamwork are essential characteristics in the upward referral process in ensuring that all parties involved in the woman’s care work together seamlessly to obtain the best results. Two domains emerged: (a) Unified trust in teamwork and (b) Collaboration with the referral hospital’s team.

*3.3.2.1 Trust in teamwork*

Unified trust in teamwork emerged as a key element for effective upward referral of obstetric emergencies. This means that the team members trust each other’s abilities and are willing to support one another during emergencies, reflecting a shared confidence. The following participants excerpts support this claim:

“ *Unity is very important for making this referral process easier, if I know that I can rely on my colleagues during emergencies; and that we trust each other’s judgment to make quick, sound and safe decisions, all is good”* (FGD2_MW2, CHC 2).

*“It’s that trust in each other’s skills that will keep the whole team moving seamlessly during such emergencies, even when we are under pressure”* (FGD4_MW4, Hospital 2).

The data sources further highlighted the significance of good working relationships and mutual trust among team members to ensure a smooth upward referral process for women with obstetric emergencies in the following excerpts:

*“… We have to all trust each other to do our part”* (FGD10_MW3, Hospital 3).

*“When there’s no formal platform where we regularly engage with them (paramedics), the issues just build up. We’re all supposed to be part of the same team”* (FGD11_MW1, Hospital 1).

*3.3.2.2 Collaboration between referral and receiving hospital teams*

Effective collaboration between the referral and receiving hospitals is yet another key characteristic in ensuring the effective upward referral of obstetric emergencies. The participants reflected on the beneficial collaborative relationships with specific hospitals, particularly in urgent cases:

*“… the collaboration between us (CHCs) and the receiving team is important, particularly for obstetric emergencies …. it is important that we try by all means to avoid unnecessary delays”* (FGD4_MW3, CHC 4).

*“Collaboration also involves working hand in hand with paramedics as the patient’s condition evolves during transit.* *It makes sure that the mother’s stable and safe*…” (FGD7_MW1, Hospital 2).

*“The key is coordination at every level, from the CHC to the receiving hospital for smooth handovers”* (IDI7_MO7).

Furthermore, the value of a collaborative relationship with MOs in critical cases was noted:

“ *When the referral comes from our manager, things move more better*” (FGD5_MW1, CHC 5).

*“For it (upward referral) to be successful, it relies on more fluid teamwork between the hospitals, doctors and midwives”* (IDI1_MO1)

Although the data sources agreed on trust and collaboration as the cornerstones for effective upward referral, some alluded to the systemic factors which often hinder these dynamics. Fragmented healthcare systems and hierarchical structures were described as key intervening conditions emerged, which created silos and undermined collaboration. This is what the participants said:

*“Collaboration is very important in such emergencies, but the healthcare system has systemic challenges. Higher ups don’t list to us, and this disconnect creates delays care for patients”* (FGD4_MW4, CHC 4).

*“The hierarchical structure makes it challenging to collaborate. Managers are working alone and do not understand some of our urgent needs”* (FGD6_MW2, Hospital 1).

*3.3.3 Effective referral outcomes*

Achieving favourable outcomes when referring obstetric emergencies to specialised care emerged as yet another characteristic for effective upward referral of obstetric emergencies. Under this attribute, two key categories emerged (a) Safe and stable transfer of the patient and (b) Ensuring timely arrival at the referral hospital. However, the extent to which these outcomes are realised is often influenced by systemic and contextual constraints that shape how effectively safety protocols and timeliness are enacted in real-world settings.

*3.3.3.1 Safe and stable transfer of the patient*

Successful transfer of obstetric emergencies is characterised by safe and stable transfer of patients which ensures that the patient is transferred in a condition that minimises risk and prevents further complications, as seen below:

*“Ensuring the patient’s safety and stability during transfer is our top priority. This involves stabilising the patient before transport and continuously monitoring them throughout the journey to minimise risks …”* (IDI11_PARAM3).

“ *We have to make sure that the patient is stable, and safe as we refer them”* (FGD6_MW4, Hospital 1).

*“If the patient is monitored during transport, that makes the whole process safer”* (FGD10_MW4, Hospital 3).

*3.3.3.2 Ensuring timely arrival at the referral hospital*

Timely arrival at the referral hospital emerged as another feature of successful upward referral of obstetric emergencies. This outcome, however, is heavily shaped by external operational factors, particularly the availability and responsiveness of ambulance services. Below are verbatim extracts from participants:

*“The success partially relies on the patient arriving here* [receiving hospital] *stable and without further complications”* (FGD11_MW3, Hospital 1).

*“The focus is not only getting the mother there on time, so the quicker the ambulance comes, the better the outcomes”* (FGD2_MW4, CHC 2).

*3.3.4 Post feedback and continuous improvement*

Post Feedback and continuous improvement emerged as another vital characteristic of effective upward referral of obstetric emergencies. The goal is to continually improve the process by discussing and receiving feedback on what went well and suggestions for improvement. Two sub-attributes were identified: (a) Post Feedback and review of referral cases for continuous improvement, and (b) Opportunities for regular engagement and learning.

*3.3.4.1 Post feedback and review of referral cases for continuous improvement*

The data sources highlighted that effective upward referral of obstetric emergencies is marked by regular feedback and continuous review of referral cases to drive ongoing improvement. This is reflected in the participant statements:

“ *Hospitals and CHCs need to work together closely to provide feedback to identify where they can improve, to avoid us receiving the same mistakes over and over again*” (FGD12_MW3, Hospital 2).

*“And without feedback, CHCs can’t learn or improve. We need a standardised back-referral process and designated liaison staff for follow-up”* (FGD12_MW4, Hospital 2).

Some participants suggested that feedback can be provided during perinatal meetings, where cases are reviewed, and constructive input is shared.

“ *Perinatal meetings are a good platform to review complex cases”* (FGD8_MW3, Hospital 2).

*“Feedback from these meetings helps improve handling of future referrals”* (FGD1_MW3, CHC 1).

“ *We need regular feedback loops*” (IDI4_MO4).

However, structural limitations were reported to compromise the usefulness of these processes. Participants noted that perinatal meetings were often attended by OMs rather than those directly involved in patient care, creating a disconnect between the review process and frontline realities.

*“My issue is that these perinatal meetings are often attended by OMs who are not actively involved in patient care. Their feedback doesn’t always reflect the real challenges we face on the ground”* (FGD4_MW2, CHC 4).

*“We are the ones who handle emergencies, but we don’t get an opportunity to discuss these issues with decision-makers. Our challenges are filtered and the feedback diluted”* (FGD13_MW1, Hospital 3).

Although feedback mechanisms are widely recognised as critical for learning and quality improvement, hierarchical communication structures and the exclusion of frontline staff frequently limit their relevance and impact.

## 4. Discussion

This study delved into healthcare workers’ perspectives of effective upward referral for women experiencing obstetric emergencies from CHCs to higher-level care within the OR Tambo district, identifying the antecedents, attributes that determine its effectiveness. However, the ability to implement these components consistently was often disrupted by broader systemic and contextual factors.

### 4.1 Antecedents for effective upward referral of obstetric emergencies

Participants identified the provision of relevant, accurate, and concise patient information and the prompt relay of vital details as a cornerstone of effective upward referral. Communication practices were viewed as essential to ensuring the preparedness of the receiving facility, avoiding misunderstandings, and preventing delays in emergency care. These findings align with broader evidence highlighting communication as a cornerstone of effective emergency referral systems. ^28^ However, intervening conditions such as poor network connectivity and unpaid phone bills often disrupted these processes, particularly in rural areas with fragile telecommunication infrastructure. In contrast to the findings of the current study, where delays persisted despite referral efforts, studies in other contexts have shown improved outcomes. For example, in Uganda, telephonic communication between sending and receiving facilities during obstetric emergencies significantly reduced admission and treatment delays. ^29^ Similarly, in Ghana, direct phone communication among providers played a critical role in enhancing the referral process for obstetric emergencies. ^30^

The findings also show that effective referrals depend heavily on adequate patient preparation and complete documentation. Participants from all cadres repeatedly emphasised this antecedent. Maintaining continuity of care was thought to depend on stabilising patients before to transfer (e.g., giving IV fluids). This is consistent with previous research, ^31^ who show that maternal morbidity and mortality are directly impacted by insufficient pre-referral care in obstetric emergencies. However, in this study, structural limitations such as ongoing midwife shortages and excessive multitasking, often compromised the ability to adequately prepare patients. Similar findings were reported, showing that structural issues impair patient record quality and hinder continuity between referral levels. ^32^

Additionally, thorough patient preparation and documentation were widely recognised by participants as essential steps in the referral process. However, these tasks were often hindered by systemic challenges such as chronic staff shortages, multitasking demands, and the absence of standardised tools. In several facilities, a single midwife was expected to simultaneously manage maternity, postnatal, and emergency units. These institutional constraints, rather than gaps in clinical knowledge or professional commitment, undermined the ability to fully stabilise patients or communicate clinical histories during handover.

Inadequate pre-referral stabilisation has been shown to increase maternal and neonatal morbidity and mortality, especially in resource-limited settings. ^32^ Participants’ reports align with existing evidence that excessive workloads and understaffing restrict midwives’ capacity to initiate critical pre-referral interventions. These gaps in preparation can delay treatment at the receiving facility, as clinicians must repeat or begin stabilisation efforts that should have been initiated at the primary care level. ^33^

The availability of resources, particularly emergency transport and ambulances emerged as a critical antecedent influencing the promptness and safety of upward referrals. The main barriers to safe transfers, according to the participants, are unresponsive emergency services, inadequately prepared ambulances and ongoing ambulance shortages. These results support the systematic review by Banchani and Tenkorang, ^34^ which identifies transportation obstacles as a major cause of maternal mortality in low-income nations. Rural ambulance delays are also considered a public health emergency, as noted by Ogunleye et al. ^35^ Notably, this study also showed that, even in cases where ambulances were available, patient safety during transit was jeopardised by subpar equipment (such as outdated monitors) and poor vehicle maintenance, pointing to a more serious systemic problem than just availability.

### 4.2 Attributes of effective upward referral of obstetric emergencies

Timely and decisive decision-making was consistently identified by participants as a defining attribute of effective upward referral. They described the urgency with which referral decisions must be made in obstetric emergencies to avoid life-threatening delays. In their view, hesitation or delayed action could compromise maternal and foetal outcomes. This aligns with findings from Curtin et al. ^13^ and Tiruneh et al., ^36^ who report that delays in clinical decision-making are closely linked to adverse maternal outcomes. Ameh ^37^ further emphasises the value of experience and training in enabling prompt action during emergencies. The study data suggest that the effectiveness of upward referral depends not only on systems and resources but also on the clinician’s ability to assess, decide, and act swiftly. As such, timely decision-making is both a defining attribute and a practical necessity in the upward referral of obstetric emergencies.

Effective referrals were also made possible by the team members’ mutual trust. Participants indicated that under high-stress situations, trust served as a mechanism to lessen hesitation and needless repeating of activities. These observations support previous observations ^38^ that found that enhanced intra-team trust is associated with better communication and efficiency during medical emergencies. Similarly, Harris et al. ^39^ emphasise that mutual trust and cohesive collaboration have a direct impact on maternal safety in obstetric facilities. For practitioners operating in an environment of urgency, uncertainty and shortage, trust was more than just interpersonal in the setting of this study; it was a coping strategy.

In addition to internal trust, participants highlighted the need for stronger collaboration between referring CHCs and receiving hospitals. Effective upward referral was often undermined by a lack of communication and continuity across facilities. Participants reported that collaborative relationships, such as joint case reviews and open communication channels, improved mutual understanding and streamlined patient transitions. This finding aligns with authors, ^40^ who argue that interprofessional collaboration in obstetric care leads to safer and more consistent referrals, especially in LMICs. Yet participants noted that where such collaboration was weak or inconsistent, delays and mismanagement were more likely to occur, negatively impacting patient outcomes. When collaboration and teamwork were present, referral processes tended to run more smoothly. However, organisational barriers, particularly rigid hierarchies and poor inter-facility coordination, limited the extent to which healthcare workers could function as a unified team across the continuum of care.

Participants consistently linked patient safety to the stability of the patient during transit and the timeliness of arrival at the receiving facility. However, they highlighted several challenges compromising safety, particularly in the rural Eastern Cape, where long distances, poorly resourced facilities, and inadequate transport infrastructure increase the risk of adverse outcomes during transfer. These reflections align with findings, ^11^ which report that geographical isolation and poor road conditions significantly delay timely referrals. Similarly, operational difficulties and remote locations hinder patient safety. ^36^ As such, safeguarding patients during inter-facility transfers is not just a logistical necessity but a core attribute of effective referral systems in obstetric emergencies.

In agreement with previous studies, ^41^ participants emphasised the importance of receiving feedback post-referrals to optimise referral systems. However, participants in this study expressed concern that such feedback often excludes frontline healthcare workers. Instead, meetings were frequently attended by senior managers, limiting opportunities for those directly involved in patient care to engage in learning and improvement processes. This contrasts with Wibbelink et al., ^42^ who advocate for inclusive feedback systems involving all levels of healthcare professionals to enhance continuity, accountability, and quality of care. Similarly, Avoka et al., ^41^ highlight that feedback and quality improvement initiatives can significantly improve outcomes in obstetric emergencies and regular reviews of referral cases help reduce the risk of adverse events. ^43^

Finally, feedback and learning were found to be inconsistent, despite participants’ clear desire for improvement. The exclusion of frontline staff from perinatal meetings not only limited the scope of review but diluted the practical insights needed to improve future referrals. As a result, opportunities for shared learning were lost, and similar challenges recurred.

Although strengthening the antecedents and attributes of upward referral is critical for improving maternal and neonatal outcomes, there are systemic barriers that effective referral practices alone cannot directly resolve. These include limited ambulance availability, poorly maintained vehicles, long distances to referral hospitals, network and communication issues and chronic staff shortages. Despite these constraints, improvements in referral practices can still provide indirect benefits. For example, thorough patient preparation and complete documentation ensure that receiving facilities can act immediately on patient arrival, even if transport is delayed. Similarly, clear communication, effective teamwork and post-referral feedback help reduce errors, support timely decision-making and create opportunities for ongoing improvement within the limitations of the existing healthcare system.

The study demonstrates that effective upward referral is conceptualised by maternity staff as a process defined by preparation, communication and coordination across levels of care. Participants viewed effective referral as contingent on specific antecedents (such as stabilisation, documentation and communication) and core attributes (such as teamwork, readiness and continuity of care). This conceptualisation positions effective referral not merely as a transfer event, but as a relational and systemic process grounded in collaborative action.

## 5. Limitations

This study provides valuable insights into the upward referral process for obstetric emergencies; however, some limitations must be acknowledged. First, the study was conducted in a single district (OR Tambo in the Eastern Cape), which may not fully capture the diversity of referral processes, healthcare infrastructures, and contextual dynamics present in other provinces. Second, the findings are based on the perceptions of healthcare workers within this specific context. While these insights are crucial, they may not be entirely transferable to urban, well-resourced, or differently structured health systems.

## 6. Recommendations

To improve the efficiency of upward referral for obstetric emergencies, there is a need to enhance network infrastructure in rural areas to guarantee reliable communication between CHCs, EMS and referral hospitals. Additionally, improving and maintaining roads leading to healthcare facilities, particularly in remote areas, is recommended to facilitate faster and safer patient transportation. By ensuring that both referring and receiving facilities have access to well-equipped ambulances specifically designated for obstetric emergencies, this helps prioritise pregnant women in need of urgent medical attention, significantly reduces waiting times and improves the efficiency of the referral system. Given that this study was conducted in a single district (OR Tambo) in the Eastern Cape, future research should explore upward referral processes across both urban and rural settings to capture regional differences in healthcare infrastructure, resource availability and referral efficiency would offer a more wide-ranging understanding of the systemic challenges and best practices in maternal healthcare referrals.

## 7. Conclusion

This study explored healthcare workers’ perspectives on the effective upward referral of obstetric emergencies from CHCs to higher-level facilities in the OR Tambo district, South Africa. It identified the critical antecedents and attributes that shape the referral process. Effective upward referral was found to hinge on timely decision-making, adequate preparation, clear communication and collaborative inter-facility relationships. However, despite healthcare workers’ knowledge and commitment, systemic and contextual challenges, such as poor communication infrastructure, staffing shortages, inadequate emergency transport, and fragmented feedback mechanisms, disrupted referral effectiveness. Addressing these challenges is vital to improving maternal and neonatal outcomes and advancing South Africa’s progress toward SDG 3.

## Ethics approval and consent to participate

Ethics approval was obtained from the Biomedical Research Ethics Committee (reference number: BREC/00006633/2024) on the 22 ^nd^ of May 2024 and all participants provided written informed consent.

## Declaration of generative AI and AI-assisted technologies in the writing process

During the preparation of this work the author used Grammarly in order to assist in reducing repetition. After using this tool/service, the author(s) reviewed and edited the content as needed and take(s) full responsibility for the content of the publication.

## Data Availability

The qualitative datasets generated and analysed during the current study are not publicly available due to ethical restrictions related to participant confidentiality and the sensitive nature of discussions around obstetric emergencies within a defined health district. Public sharing of full interview and focus group transcripts could risk indirect identification of participants and healthcare facilities. Additionally, the study is **still ongoing** and data collection and analysis continue to ensure comprehensive coverage of obstetric emergencies within the study sites. Furthermore, additional publications are planned that will draw upon these datasets, meaning unrestricted public release at this stage could compromise the integrity of future research outputs and analyses. The Biomedical Research Ethics Committee of the University of KwaZulu-Natal (BREC/00006633/2024) approved the study, including procedures to ensure participant confidentiality and secure handling of qualitative data. In line with these ethical requirements, raw qualitative data are not made openly accessible. De-identified excerpts of the data may be made available upon reasonable request for academic research purposes, subject to approval by the University of KwaZulu-Natal and compliance with ethical and data protection requirements. Requests should include a brief description of the intended use of the data and be directed to the corresponding author at finaljuqu@gmail.com. Access will only be granted where participant confidentiality can be fully maintained.
