# Supplementary material for: Understanding Antecedents and Attributes in the Context of Upward Referral for Obstetric Emergencies: A Grounded Theory Study
Source: F1000Res. 2026 Mar 2;15:22. Originally published 2026 Jan 7. [Version 3] doi: 10.12688/f1000research.174876.3 (PMC13594606; doi:10.12688/f1000research.174876.3)
Supplement: Peer review history

# Reviewer report: Reviewer response for version 3

**DOI:** 10.5256/f1000research.197055.r463708

**Published:** 2026-09-21

**Authors:** Evie Kendal

**Affiliations:** 1 Swinburne University of Technology, Melbourne, Australia

My comments have been appropriately addressed. Thank you for providing this important research perspective.

Is the work clearly and accurately presented and does it cite the current literature?

Yes

If applicable, is the statistical analysis and its interpretation appropriate?

Yes

Are all the source data underlying the results available to ensure full reproducibility?

No

Is the study design appropriate and is the work technically sound?

Yes

Are the conclusions drawn adequately supported by the results?

Yes

Are sufficient details of methods and analysis provided to allow replication by others?

Partly

Reviewer Expertise:

Reproductive bioethics, epidemiology, public health

I confirm that I have read this submission and believe that I have an appropriate level of expertise to confirm that it is of an acceptable scientific standard.

# Reviewer report: Reviewer response for version 2

**DOI:** 10.5256/f1000research.196682.r460625

**Published:** 2026-02-23

**Authors:** Qudus Lawal

**Affiliations:** 1 Irrua Specialist Teaching hospital, Irrua, Nigeria

Satisfactory response

Is the work clearly and accurately presented and does it cite the current literature?

Yes

If applicable, is the statistical analysis and its interpretation appropriate?

Not applicable

Are all the source data underlying the results available to ensure full reproducibility?

Yes

Is the study design appropriate and is the work technically sound?

Yes

Are the conclusions drawn adequately supported by the results?

Yes

Are sufficient details of methods and analysis provided to allow replication by others?

Yes

Reviewer Expertise:

Obstetrics and gynaecology

I confirm that I have read this submission and believe that I have an appropriate level of expertise to confirm that it is of an acceptable scientific standard.

# Reviewer report: Reviewer response for version 1

**DOI:** 10.5256/f1000research.192816.r452022

**Published:** 2026-02-19

**Authors:** Evie Kendal

**Affiliations:** 1 Swinburne University of Technology, Melbourne, Australia

Thank you for the opportunity to review this work, which examines barriers to achieving a maternal continuum of care through effective referrals in OR Tambo, Eastern Cape. Some minor comments:

 1) The phrasing of "A significant portion of nearly two-thirds of these fatalities occur in Sub-Saharan Africa (SSA)" is ambiguous, as it is unclear what the 2/3 is referring to.

 2) It is unclear how authors dealt with disagreements during coding - was there a tie breaker method? How many authors agreed on the codes?

 3) It would be beneficial to consider the barriers to care that effective referrals themselves are unlikely to influence directly, and how some indirect benefit might be extracted.

Is the work clearly and accurately presented and does it cite the current literature?

Yes

If applicable, is the statistical analysis and its interpretation appropriate?

Yes

Are all the source data underlying the results available to ensure full reproducibility?

No

Is the study design appropriate and is the work technically sound?

Yes

Are the conclusions drawn adequately supported by the results?

Yes

Are sufficient details of methods and analysis provided to allow replication by others?

Partly

Reviewer Expertise:

Epidemiology, maternal health in LMICs, bioethics

I confirm that I have read this submission and believe that I have an appropriate level of expertise to confirm that it is of an acceptable scientific standard, however I have significant reservations, as outlined above.

# Response

**Published:** 2026-02-23

**Authors:** Final Zimkhitha Juqu

**Affiliations:** College of Health Sciences, University of KwaZulu-Natal School of Health Sciences, Durban, KwaZulu-Natal, South Africa

We thank the reviewer for the thoughtful and constructive comments, which have helped to strengthen the clarity and depth of the manuscript. We respond to each point below.

 Comment 1: We agree that the phrasing was ambiguous. The sentence has been revised in the next version to clearly specify that approximately two-thirds of *global maternal deaths* occur in Sub-Saharan Africa.

 Comment 2: The data analysis section has been expanded in the revised version to describe how coding disagreements were managed through team discussion and consensus.

 Comment 3: The Discussion section has been strengthened to acknowledge barriers to maternal care that may not be directly influenced by referral effectiveness and to reflect on how indirect benefits may nonetheless be derived.

# Reviewer report: Reviewer response for version 1

**DOI:** 10.5256/f1000research.192816.r450584

**Published:** 2026-02-12

**Authors:** Qudus Lawal

**Affiliations:** 1 Irrua Specialist Teaching hospital, Irrua, Nigeria

The manuscript is well written, and it uses appropriate methodology for the study.

 The use of theoretical sampling is particularly appropriate to get in-depth knowledge and follow leads from the codes and patterns generated.

 The following minor concerns should be addressed:

*1 **) "FGDs were conducted with midwives at 5 CHCs and hospitals. A total of 13 FGDs were conducted: five at CHCs and eight at the hospitals"***

 This should be revised to ensure clarity and address the different numbers mentioned.

***2) Data were collected from August 2024 to May 2025, using focus group discussions (FGDs), individual in-depth interviews (IDIs), document analysis, and observations***

 This statement was repeated twice

***3)  "involved three to 5 midwives, as recommended."***

 Change 5 to five

Is the work clearly and accurately presented and does it cite the current literature?

Yes

If applicable, is the statistical analysis and its interpretation appropriate?

Not applicable

Are all the source data underlying the results available to ensure full reproducibility?

Yes

Is the study design appropriate and is the work technically sound?

Yes

Are the conclusions drawn adequately supported by the results?

Yes

Are sufficient details of methods and analysis provided to allow replication by others?

Yes

Reviewer Expertise:

Obstetrics and gynaecology

I confirm that I have read this submission and believe that I have an appropriate level of expertise to confirm that it is of an acceptable scientific standard.

# Response

**Published:** 2026-02-16

**Authors:** Final Zimkhitha Juqu

**Affiliations:** College of Health Sciences, University of KwaZulu-Natal School of Health Sciences, Durban, KwaZulu-Natal, South Africa

Comment 1: Thank you for highlighting this. The sentence will be revised in the next version to clearly specify the total number of FGDs conducted and their distribution between CHCs and hospitals to avoid any ambiguity.

 Comment 2: We appreciate the reviewer’s attention to detail. The duplicated statement will be removed in the revised version of the manuscript.

 Comment 3: We appreciate the reviewer’s attention to detail. This will be changed in the revised version of the manuscript.
